# Amylin Protein Expression in the Rat Brain and Neuro-2a Cells

**DOI:** 10.3390/ijms23084348

**Published:** 2022-04-14

**Authors:** Yeong-Min Yoo, Eui-Man Jung, Eui-Bae Jeung, Bo Ram Jo, Seong Soo Joo

**Affiliations:** 1 East Coast Research Institute of Life Science, College of Life Science, Gangneung-Wonju National University, Gangneung 25457, Korea; 2 Department of Molecular Biology, College of Natural Sciences, Pusan National University, Busan 46241, Korea; jungem@pusan.ac.kr; 3 Laboratory of Veterinary Biochemistry and Molecular Biology, College of Veterinary Medicine, Chungbuk National University, Cheongju 28644, Korea; ebjeong@chungbuk.ac.kr; 4 Department of Marine Bioscience, College of Life Science, Gangneung-Wonju National University, Gangneung 25457, Korea; boram0430@nate.com

**Keywords:** amylin, 17β-estradiol, progesterone, dexamethasone, rat brain, Neuro-2a

## Abstract

The localization and expression of amylin protein in the rodent brain and mouse neuroblastoma Neuro-2a (N2a) are less widely known. Thus, this study investigated the expression distribution of amylin in the rat brain and N2a treated with steroid hormones. Amylin protein was identified in the olfactory bulb, cerebral cortex, dentate gyrus, thalamus, hypothalamus, ventral tegmental area (VTA), cerebellum, and brain stem in the rat brain. Additionally, the amylin protein was localized with the mature neurons of the cerebral cortex and dopaminergic neurons of the VTA. Progesterone (P4) and dexamethasone (Dex) significantly decreased, and 17β-estradiol (E2) increased the amylin protein level in the cerebral cortex. The P4 receptor antagonist RU486 significantly influenced the effects of P4 and Dex, and the E2 receptor antagonist ICI 182,780 slightly changed E2′s effect. Amylin protein expression was significantly reduced in the VTA by P4 and Dex, and its expression was changed only following P4 plus RU486 treatment. It was confirmed for the first time that amylin protein is strongly expressed in the cytoplasm in N2a cells using immunofluorescent staining. P4 increased the levels of amylin, and RU486 treatment decreased them. Dex significantly increased the levels of amylin protein. RU486 treatment reversed the effects of Dex. Therefore, amylin protein is expressed in the cerebral cortex neurons and dopaminergic neurons of the VTA of the immature rat brain. P4 and Dex influence the expression of amylin protein in the rat brain and N2a cells.

## 1. Introduction

Amylin or islet amyloid polypeptide (IAPP) has been established as the main constituent of pancreatic islet amyloid deposits in type 2 diabetics and diabetic cats independently by Westermark et al. [1] and Cooper et al. [2]. Amylin is a 37-amino acid peptide synthesized and secreted with insulin in the pancreatic β cells that activates G protein-coupled receptor signaling [3]. Amylin is a circulating glucoregulatory hormone that regulates energy homeostasis in rodents [3,4]; it is involved in the cardiovascular system and bone metabolism [3,5]. Amylin acts primarily in the periventricular organs of the rat brain and functionally interacts with leptin and estradiol [3].

Glucocorticoid and progesterone (P4) are steroid hormones that are classically known to mediate distinct physiological functions. Glucocorticoids or dexamethasone (Dex) regulate glucose metabolism, inflammation inhibition, and immune function [6]. Both glucocorticoid and P4 have been shown to stimulate or inhibit the growth of breast cancer cells [7,8,9,10,11]. Rat growth hormone and Dex treatment of transgenic mice carrying human amylin induce amylin-derived islet amyloid and islet dysfunction [12]. Estradiol (E2) is an estrogen steroid and a primary female sex hormone. It is currently unclear whether E2 regulates the effects of amylin in the brainstem or hypothalamic structures [3]. E2 administration into male transgenic mice carrying human amylin decreased islet amyloid aggregates and prevented progression to pancreatic β-cell failure [13].

However, the localization and expression of amylin protein in the rodent brain are less widely known. Thus, this study investigated the amylin distribution pattern in the rat brain. Additionally, to identify the influence of steroid hormones on the expression of amylin protein in the brain, immature female rats were injected for 5 days with E2, P4, Dex, and their antagonists (ICI 182,780 and RU486). This study investigated the expression of amylin protein following P4 and Dex treatment of mouse neuroblastoma Neuro-2a (N2a).

## 2. Results

### 2.1. Expression of Amylin Protein in Immature Rat Brain Regions

**Figure 1:**
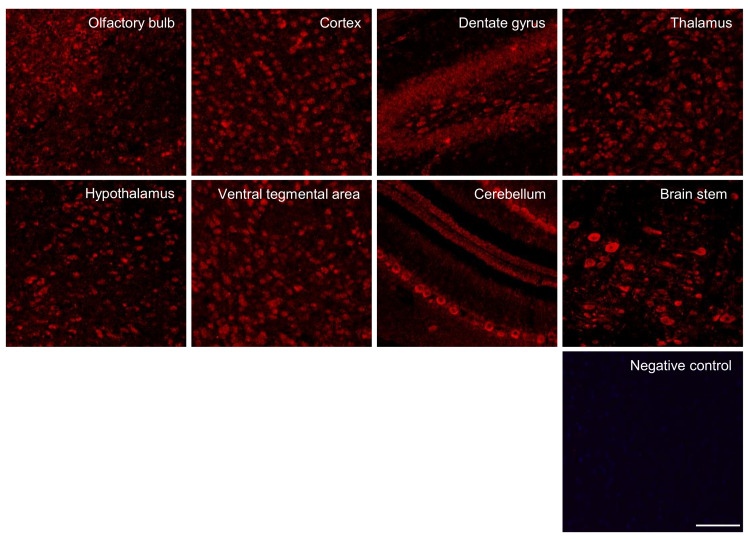
Distribution of amylin in the immature rat brain. Samples from different regions of the olfactory bulb, cerebral cortex, dentate gyrus, thalamus, hypothalamus, ventral tegmental area, cerebellum, and brain stem were analyzed in immature rat brains. Fluorescence photomicrographs showed amylin in a sagittal section. Amylin is expressed widely in the brain. Scale bar, 50 μm.

Amylin protein was identified via immunofluorescent staining of the immature rat brain. Amylin-positive cells were found in the olfactory bulb, cerebral cortex, dentate gyrus, thalamus, hypothalamus, ventral tegmental area (VTA), cerebellum, and brain stem (Figure 1).

### 2.2. Colocalization of Amylin Protein with Neuron-Specific Markers in the Immature Rat Brain

**Figure 2:**
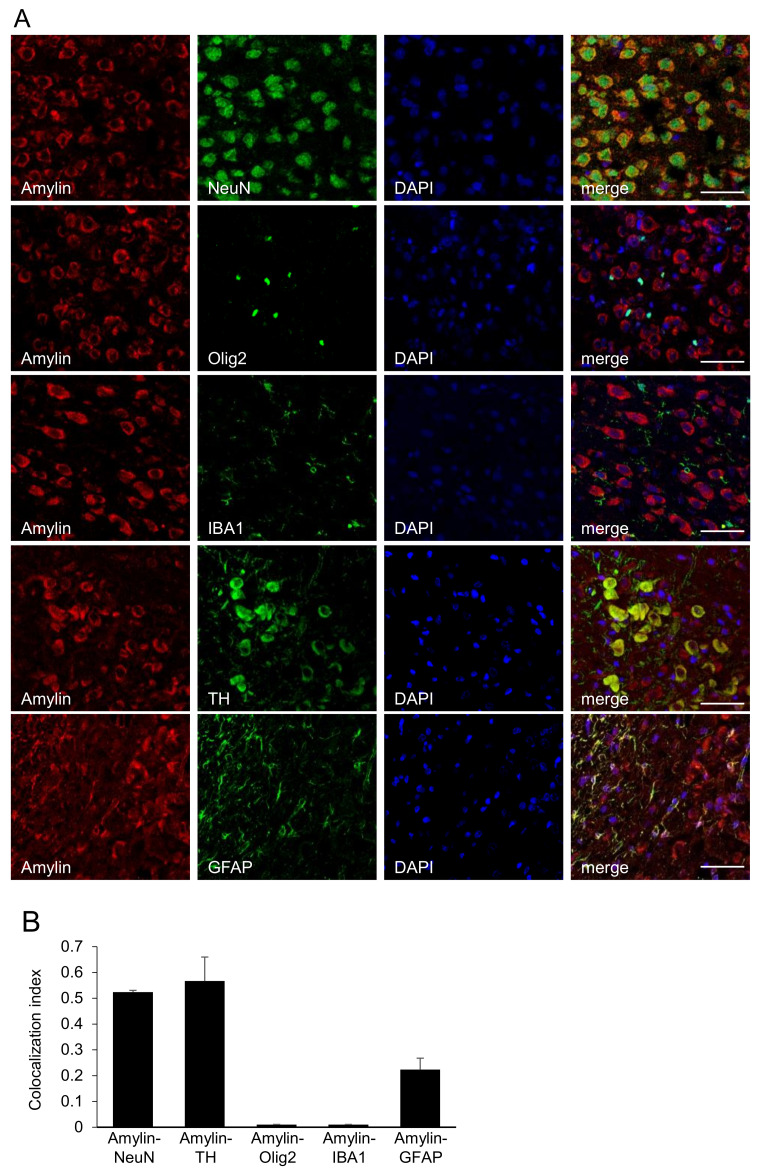
Colocalization of amylin protein with neuron-specific markers in the immature rat brain. (**A**) Amylin protein was co-expressed with neuron-specific markers such as NeuN, a marker of mature neurons; TH, a marker of dopaminergic neurons; IBA1, a marker of microglia; olig2, a marker of oligodendrocytes; and GFAP, a marker of astrocytes. Amylin protein was colocalized with mature neurons in the cerebral cortex and dopaminergic neurons in the VTA. (**B**) Quantification of colocalization in the immature rat brain. Colocalization was shown in yellow in the merged image. Colocalization analysis was performed using ImageJ software. Scale bar, 50 μm.

We performed double immunofluorescence staining to determine whether amylin protein co-expressed with neuron-specific markers such as NeuN (a marker of mature neurons), tyrosine hydroxylase (TH, a marker of dopaminergic neurons), IBA1 (a marker of microglia), olig2 (a marker of oligodendrocytes), and GFAP (a marker of astrocytes). Amylin protein was colocalized with mature neurons in the cerebral cortex and dopaminergic neurons in the VTA (Figure 2).

### 2.3. Amylin Protein Expression by Steroid Hormones in Immature Rat Brain Regions

**Figure 3:**
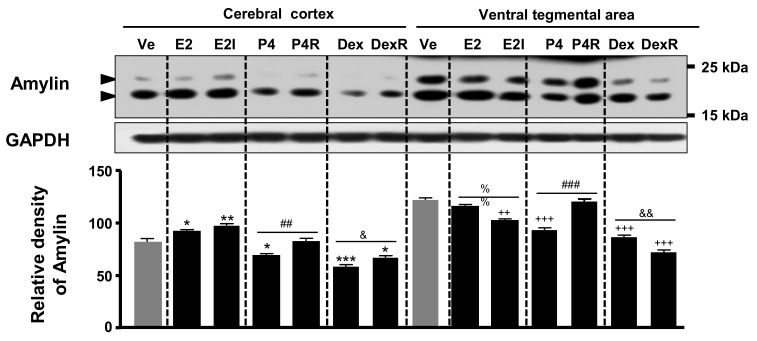
Amylin protein expression changes induced by steroid hormones in immature rat brain regions. Immature SD rats (postnatal days 10–14) were subcutaneously injected with 17β-estradiol (E2, 50 μg/kg body weight (BW)), dexamethasone (Dex, 10 mg/kg BW), progesterone (P4, 20 mg/kg BW), or vehicle (corn oil; Sigma-Aldrich) once per day for 5 consecutive days. ICI 182,780 (10 mg/kg BW) and mifepristone (RU486, 50 mg/kg BW) were treated 30 min before hormone administration. Amylin protein was then detected by Western blotting. The relative amounts of proteins were quantified as described in Section 4. Data are presented as the mean ± standard error of three experiments. * *p* < 0.05, ** *p* < 0.01, *** *p* < 0.001 vs. the control of cerebral cortex; ++ *p* < 0.01, +++ *p* < 0.001 vs. the control of the VTA; ^%%^ *p* < 0.01, E2 vs. E2 + ICI; ^##^ *p* < 0.01 and ^###^ *p* < 0.001, P4 vs. P4 + RU486; ^&^ *p* < 0.05 and ^&&^ *p* < 0.01, Dex vs. Dex + RU486. Ve, vehicle; E2, 17β-estradiol; P4, progesterone, Dex dexamethasone; I, ICI 182,780; R, RU486.

Immature female rats were injected for 5 days with E2, P4, Dex, and their antagonists (ICI 182,780 and RU486) to investigate the expression of amylin protein in the cerebral cortex and VTA. The amylin protein level was significantly decreased by P4 and Dex in the cerebral cortex but increased by E2. Additionally, RU486 significantly increased the effects of P4 and Dex, and ICI 182,780 slightly changed E2′s effects (Figure 3). In the VTA, the amylin protein expression was significantly decreased by P4 and Dex but was not influenced by E2; its expression was significantly reversed in P4 plus RU486 treatment, E2 plus ICI 182,780, or Dex plus RU486 (Figure 3).

### 2.4. Amylin Protein and ERK Expression Following P4 Treatment of N2a Cells

**Figure 4:**
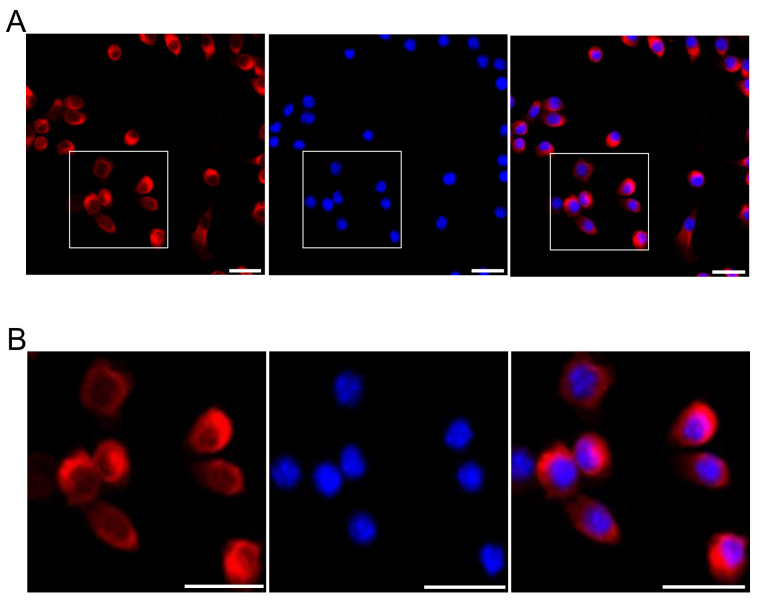
Amylin protein expression in the cytoplasm of N2a cells (**A**). Box indicates the magnified area (**B**). Immunofluorescent staining was described in Materials and Methods. Scale bar, 20 μm.

**Figure 5:**
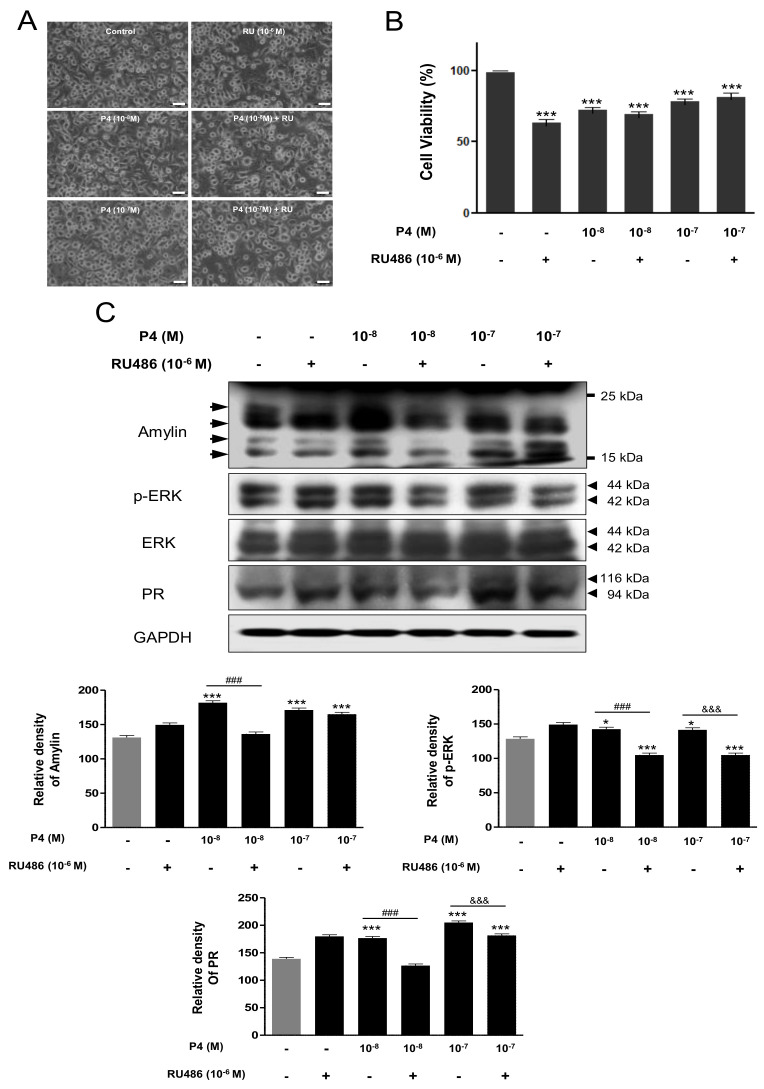
Amylin protein and ERK expression changes induced by P4 treatment in N2a cells. N2a cells were cultured in DMEM with 10% FBS, 100 µg/mL streptomycin sulfate, and 100 U/mL penicillin G sodium at 37 °C and 5% CO_2_. Cells treated with P4 (10^−7^, 10^−8^ M) with/without antagonist (RU486, 10^−6^ M) for 24 h. N2a cell morphology (**A**). Cell viability (**B**). Western blotting detected amylin, p-ERK, and progesterone receptor (PR) (**C**). The relative amounts of proteins were quantified as described in Section 4. Data were presented as the mean ± standard error of three experiments. * *p* < 0.05 and *** *p* < 0.001 vs. the control; ^###^ *p* < 0.001 and ^&&&^ *p* < 0.001, P4 vs. P4 + RU486. Scale bar, 25 μm.

First, amylin protein was identified via immunofluorescence staining in N2a cells. Amylin protein was strongly expressed in the cytosol of N2a cells (Figure 4). To confirm the expression of amylin protein under P4, mouse neuroblastoma N2a cells were treated with P4 (10^−8^, 10^−7^ M) with and without RU486 (10^−6^ M) for 24 h. We detected no significant difference in cell morphology with P4 treatment combined with or without RU486 compared with the control; however, cell viability decreased significantly following treatment with P4 combined with or without RU486 compared with the control (Figure 4B and Figure 5A). The levels of amylin protein, p-ERK, and progesterone receptor (PR) were significantly increased by P4 treatment compared with the control. P4 plus RU486 treatment decreased the levels of amylin protein, p-ERK, and PR compared with P4 alone—with the exception of amylin protein expression following exposure to 10^−7^ M P4 plus RU486 (Figure 5C).

### 2.5. Amylin Protein and ERK Expression Following Dex Treatment of N2a Cells

**Figure 6:**
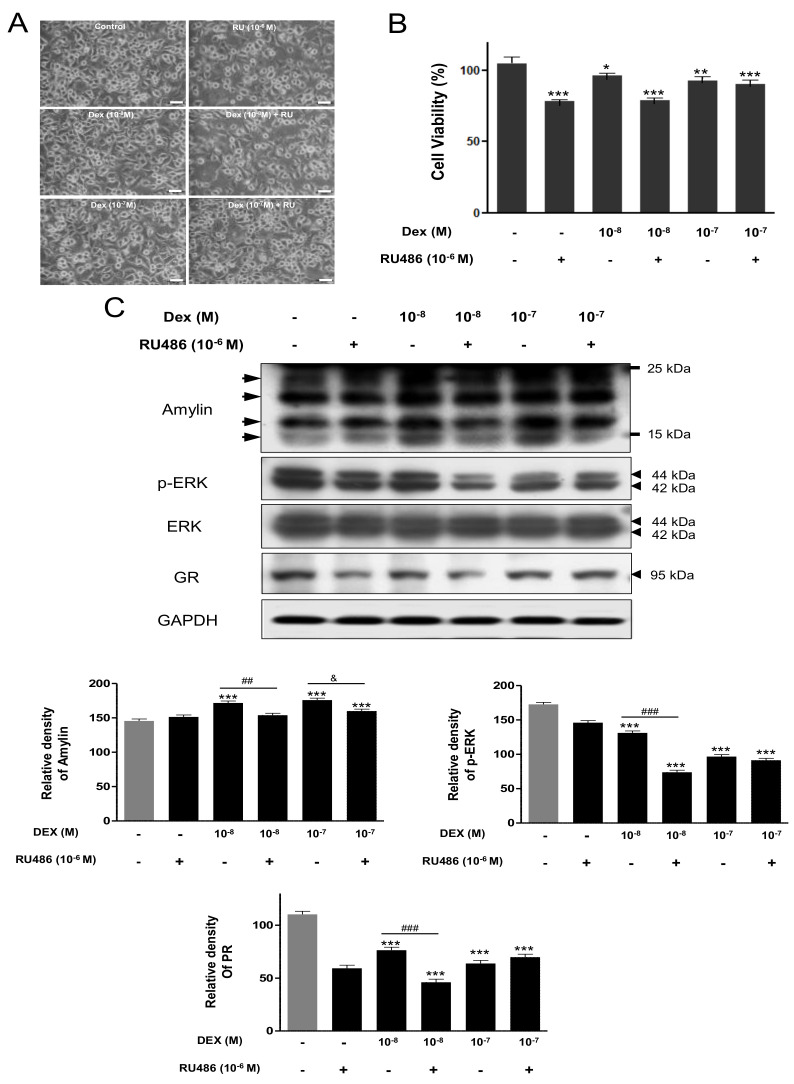
Amylin protein and ERK expression changes induced by Dex treatment in N2a cells. N2a cells were cultured in DMEM with 10% FBS, 100 µg/mL streptomycin sulfate, and 100 U/mL penicillin G sodium at 37 °C and 5% CO_2_. Cells treated with Dex (10^−7^, 10^−8^ M) with/without antagonist (RU486, 10^−6^ M) for 24 h. N2a cell morphology (**A**). Cell viability (**B**). Western blotting detected amylin, p-ERK, and progesterone receptor (PR) (**C**). The relative amounts of proteins were quantified as described in Section 4. Data are presented as the mean ± standard error of three experiments. * *p* < 0.05, ** *p* < 0.01 and *** *p* < 0.001 vs. the control; ^##^ *p* < 0.01, ^###^ *p* < 0.001 and ^&^ *p* < 0.05, P4 vs. P4 + RU486. Scale bar, 25 μm.

To confirm the expression of amylin protein under Dex, N2a cells were treated with Dex (10^−8^, 10^−7^ M) with or without RU486 (10^−6^ M) for 24 h. There was no significant difference in cell morphology following Dex treatment with or without RU486 compared with the control; however, cell viability decreased significantly by Dex combined with or without RU486 compared with the control (Figure 6A,B). The levels of amylin protein were significantly increased by Dex treatment compared with the control, and RU486 treatment reversed the effect of Dex. P4 treatment decreased the expression of p-ERK and glucocorticoid receptor (GR) significantly compared with the control. Treatment with P4 (10^−8^ M) plus RU486 decreased the levels of p-ERK and GR compared with Dex treatment alone—however, this did not occur with treatment with 10^−7^ M P4 plus RU486 (Figure 6C).

## 3. Discussion

Although the production of amylin (or amylin-like peptides) in the brain has long been suggested [14,15,16], the properties of each detected peptide have been unclear, partly due to the lack of specific detection techniques for distinguishing amylin from structurally related calcitonin gene-related peptides. A few studies have suggested that the brain produces amylin [17,18].

First, amylin is expressed in the endocrine cells of the gastrointestinal tract [19] and the dorsal root ganglia and spinal cord [20], as well as in the neurons located in the lateral hypothalamus, arcuate nucleus (ARC), paraventricular (PVN), and dorsomedial hypothalamic nuclei (DMN) of mice [17]. In particular, the expression of amylin in the hypothalamus appears to vary depending on the metabolic state, and the expression of amylin in females is higher than in male mice, indicating sex differences. Second, a 24-fold increase in amylin mRNA expression has been reported in the medial preoptic area of the hypothalamus in lactating mother rats but not in male rats [18]. This increase of amylin expression in the medial preoptic area, as the significant brain area mediating maternal behaviors, suggests a role for central amylin in maternal adaptation [21,22]. Third, Boccia et al. [23] recently reported that amylin is centrally produced by neurons of the medial preoptic nucleus (MPO), medial preoptic area (MPA), and multiple nuclei of the hypothalamus, including the ARC and area subpostrema (ASP). Amylin activates downstream pathways in the area postrema (AP), which reduces food intake [23,24]. Additionally, increased energy expenditure has been observed in rats after acute central injection of amylin into the third cerebral ventricle or AP [25,26]. Amylin acts directly on pro-opiomelanocortin (POMC) and neuropeptide Y (NPY) neurons to maintain energy homeostasis [23,27]. Lastly, D’Este et al. [28] have reported that amylin is involved in the somatic and visceral sensory function of discrete areas in the central nervous system of male rats based on immunohistochemical analysis; they demonstrated the presence of amylin-immunoreactive nerve fibers in the isolated location, the AP, and in some neuronal cell bodies of other sensory nuclei. A network of TH-immunoreactive nerve fibers often surrounds amylin-immunoreactive cell bodies.

The present study demonstrated amylin protein expression in the brain using immunofluorescence staining of 80-μm-thick coronal and sagittal sections obtained with a vibratome. Thus, amylin protein was identified in the olfactory bulb, cerebral cortex, dentate gyrus, thalamus, hypothalamus, VTA, cerebellum, and brain stem in the immature rat brain via immunofluorescent staining (Figure 1). Amylin protein was colocalized with mature neurons in the cerebral cortex via NeuN staining and with dopaminergic neurons in the VTA via TH staining (Figure 2).

Estrogen influences the basal forebrain of the rat and regulates the cholinergic neurons that project into the cerebral cortex and hippocampus involved in cognitive enhancement [29]. E2 also induces acetylcholinesterase and choline acetyltransferase (ChAT) activity, suggesting a general trophic effect on cholinergic neurons [29,30,31,32]. E2 administration prevents neurodegeneration in the brain due to declining estrogen levels during menopause [33] and blocks the actions of neurotoxic agents or inhibits their generation [34,35,36].

P4 regulates the central nervous system (CNS) and exhibits neuroprotective and cognitive effects [37]; it alleviates the inflammatory response and improves neurological function after ischemic stroke [38]. The neuronal effects of P4 are mediated by PRs in steroid-sensitive neurons [39]. PRs are expressed in the olfactory bulb, striatum, cortex, thalamus, hypothalamus, septum, hippocampus, cortex, hypothalamus, and cerebellum of the CNS [37,40]. This widespread PR distribution in the brain suggests a key role in neuroendocrine regulation, reproductive function, neuroprotection, cognition, motor, sensory function, aggression, and anxiety [40].

Glucocorticoids can protect or support normal organ function under stress following CNS injury, and a high-dose Dex treatment can decrease intracellular calcium in hypothalamic neurons [41]. Dex binds to GR, which is expressed in nearly all cell types with varying effects. Dex can prevent glutamate-induced cell death in cortical neurons by decreasing calcium signaling [42].

However, few studies have reported the effects of steroid hormones on amylin protein in the brain and neurons; however, several studies have reported the effect of amylin on islets mediated via E2 and Dex. E2 decreases amylin aggregates and prevents the decline of pancreatic β-cell function in male transgenic mice carrying human amylin [13]. Dex treatment induces amylin-derived islet amyloid and islet dysfunction in transgenic mice with human amylin [12]. Additionally, Dex treatment increases amylin levels in rat islets [20].

In this study, amylin protein expression was significantly decreased by P4 and Dex in the rat cerebral cortex and VTA. Amylin protein expression in the cerebral cortex was reversed in rats exposed to P4 and Dex combined with RU486, whereas its expression in the VTA was changed only in the animals treated with P4 plus RU486 (Figure 3). Additionally, the expression of amylin protein under P4 and Dex treatment was established in N2a cells. P4 (10^−8^ M) and Dex (10^−8^, 10^−7^ M) treatments significantly increased amylin levels, and P4 and Dex plus RU486 treatments decreased amylin levels compared with the control (Figure 4 and Figure 5).

The expression of amylin protein by P4 and Dex was significantly decreased in the cerebral cortex and VTA but significantly increased in N2a cells due to the following reasons: Immature SD rats were subcutaneously injected with E2, Dex, and P4 once daily for 5 consecutive days, whereas cells were treated with P4 (10^−7^, 10^−8^ M) and Dex (10^−7^, 10^−8^ M) with or without antagonist (RU486, 10^−6^ M) once every 24 h. Therefore, there may be a difference in the timing or mechanism of action, and P4 and Dex may reverse the amylin protein levels of rat brain and N2a cells.

Our previous study identified three amylin protein bands ranging between 15 and 25 kDa in pancreatic INS-1E cells [43,44]. The present study detected two bands of amylin oligomers ranging between 15 and 25 kDa in rat brains and three or four bands in N2a cells using an amylin antibody (LS-C352341) provided by LifeSpan BioSciences. Ly et al. [45] reported two bands of amylin protein ranging in size between 15 and 25 kDa in rat and human brain tissues using amylin (T-4157) provided by Bachem-Peninsula Laboratories. Although the antibody used by Ly et al. [46] was different from the one used in the present study, the in vivo and in vitro results showed similar patterns of amylin protein bands: oligomerized amylin protein ranging in size between 15 and 25 kDa showed three to four bands in cells and two bands in rat brain. However, Jung et al. [43] demonstrated that melatonin increases the expression or oligomerization of murine amylin under endoplasmic reticulum stress in rat INS-1E cells. Ly et al. [46] identified amylin accumulation as a trigger of brain endothelial dysfunction in diabetes-associated dementia and stroke. The present study investigated the distribution and expression of amylin protein in the rat brain and mouse neuroblastoma N2a cells with steroid hormones. Amylin colocalized with the cerebral cortex neurons and dopaminergic neurons of the VTA in the immature rat brain, and P4 and Dex influenced the expression of amylin protein in the rat brain and N2a cells. Recently, functional studies of amylin have been conducted [44,47]. Treatment of INS-1E cells with 2-deoxy-D-glucose has suggested that the relative correlation between amylin and insulin is related to human amylin toxicity in pancreatic beta cells [44]. Human amylin was overexpressed in INS-1E cells, at which point endoplasmic reticulum stress was able to induce aggregation of human amylin protein [47].

## 4. Materials and Methods

### 4.1. Animal Treatment

Immature female Sprague-Dawley (SD) rats weighing 15–20 g were obtained from Samtako (Osan, Korea). All rats were housed in polycarbonate cages and were allowed to acclimate to their housing in an environmentally controlled room (temperature 23 ± 2 °C, relative humidity 50 ± 10%, frequent ventilation, and a 12 h light/dark cycle). After approximately 7 days of acclimatization, immature SD rats (postnatal days 10–14) were separated into seven groups (n = 7) and were subcutaneously injected with 17β-estradiol (E2, 50 μg/kg body weight (BW); Sigma-Aldrich, St. Louis, MO, USA), dexamethasone (Dex, 10 mg/kg BW; Sigma-Aldrich), progesterone (P4, 20 mg/kg BW; Sigma-Aldrich), or vehicle (corn oil; Sigma-Aldrich) once per day for 5 consecutive days. Antagonist groups were treated with ICI 182,780 (10 mg/kg BW; Tocris, Avonmouth, UK) and mifepristone (RU486, 50 mg/kg BW; Sigma-Aldrich) 30 min before hormone administration. All immature rats were then euthanized with ether, and tissue samples were collected 12 h after the final injection. The Institutional Animal Care and Use Committee of Chungbuk National University approved all experimental procedures and animal treatments.

### 4.2. Cell Culture

Mouse neuroblastoma N2a cells were cultured in Dulbecco’s modified Eagle’s medium (DMEM, Invitrogen, Carlsbad, CA, USA) with 10% fetal bovine serum (FBS, Invitrogen), 100 µg/mL streptomycin sulfate, and 100 U/mL penicillin G sodium at 37 °C and 5% CO2. Cells were treated with P4 (10^−7^, 10^−8^ M) and Dex (10^−7^, 10^−8^ M) with/without antagonists (RU486, 10^−6^ M) for 24 h.

### 4.3. Cell Viability Assay

According to the manufacturer’s protocol, cell survival was determined using a Cell Counting Kit-8 (Dojindo Molecular Technologies, Inc., Rockville, MD, USA). The N2a cells were cultured in 96-well plates (Corning, Inc., Corning, NY, USA) at a density of 5 × 10^3^/well. The cells were treated with the 10 μL kit solution, and incubated for 30 min, and their absorbance was measured at 450 nm. The percentage of viable cells per sample was calculated by: Viability (%) = [(total signal-background signal)/control signal] × 100.

### 4.4. Immunofluorescence Assay of Rat Brain

The brain sections of rats were examined for immunofluorescence as described previously [48]. The rat brain was fixed with 4% paraformaldehyde, and serial 80 μm-thick coronal and sagittal sections were cut on a vibratome (Leica, VT1000S, Nussloch, Germany). Brain sections were immersed in 0.5% Triton X-100 for 15 min to improve antibody penetration and were incubated in a blocking solution (5% normal goat serum, normal donkey serum, and 0.3% Triton X-100) for 1 h. The primary antibodies used were amylin antibody (LifeSpan BioSciences, WA, USA, LS-C352341, 1:500), GABA (Abcam, Cambridge, UK, ab17413, 1:500), NeuN (Millipore, Taunton, MA, USA, MAB377, 1:500), tyrosine hydroxylase or TH (Aves, Tigard, OR, USA, TYH, 1:500), IBA1 (Abcam, ab5076, 1:500), Olig2 (Thermo Fisher Scientific, Waltham, MA, USA, MA5-15810, 1:500), GFAP(Abcam, ab53554, 1:1000); all were used for 24 h at 4 °C. Following washing in PBS, appropriate secondary antibodies conjugated with Alexa Fluor dyes (Invitrogen Corporation, CA, USA, 1:1000) were used to detect primary antibodies. DAPI (Thermo Fisher Scientific, D1306, 1:1000) was incubated to stain nuclei. Images of brain sections were taken with Zeiss LSM710 confocal microscopes (Athen, GA, USA).

Confocal images were acquired with a Zeiss LSM 880 with an Airyscan confocal laser scanning microscope in sequential mode to avoid cross-talk between channels. The same conditions were used to measure colocalization. Double-stained samples were excited with 488 nm and 594 nm light.

### 4.5. Immunofluorescence Staining of N2a Cells

N2a cells grown on culture slides (BD Falcon Labware, REF 354108) were permeabilized and fixed in methanol at −20 °C for 3 min. Cells were washed with phosphate-buffered saline (PBS), blocked with 10% bovine serum albumin (Sigma-Aldrich) in PBS for 10 min, and incubated with the primary antibody in blocking buffer for 1 h at room temperature (RT). Cells were hybridized with secondary antibodies for 1 h at RT. The coverslips were mounted on glass slides using Vectashield mounting medium (Vector Labs Inc., Burlingame, CA, USA). The primary antibody was the amylin antibody (catalog no. LS-C352341; 1:500), and the secondary antibody was Alexa 594 (red)-conjugated anti-rabbit IgG (Vector Laboratories Inc., Burlingame, CA, USA). Cells were stained with DAPI for 10 min.

### 4.6. Western Blot Analysis

Western blotting was performed following the methods reported by Yoo et al. [45]. Cells was prepared using a buffer containing 150 mM NaCl, 1% NP-40, 50 mM Tris-HCl, (pH 7.4), 0.1 mM phenylmethylsulfonyl fluoride, 5 µg/mL aprotinin, 5 µg/mL pepstatin A, 1 µg/mL chymostatin, 5 mM Na3VO4 and 5 mM NaF. Protein concentration was determined using the BCA assay (Sigma-Aldrich; Merck KGaA). Proteins (40 µg) were separated by 12% SDS-PAGE and then transferred to PVDF membranes (Sigma-Aldrich; Merck KGaA). The PVDF membranes were blocked with 5% non-fat dry milk (Santa Cruz Biotechnology, Inc., Dallas, TX, USA) in TSB-0.001% Tween 20 (Sigma-Aldrich; Merck KGaA) for 1 h at room temperature and then incubated with the following primary antibodies overnight at 4 °C: The amylin antibody (catalog no. LS-C352341; 1:1000) was provided from LifeSpan BioSciences, Inc. Phospho-ERK (catalog no. sc-7380; 1:500), ERK (catalog no. sc-93; 1:500), progesterone receptor (PR, catalog no. sc-538; 1:500), glucocorticoid receptor (GR, catalog no. sc-393232; 1:500), and GAPDH (catalog no. sc-25778; 1:500) were obtained from Santa Cruz Biotechnology, Inc. Subsequently, the membranes were incubated with anti-mouse IgG (catalog no. 7076; 1:1000; Cell Signaling Technology, Inc., Danvers, MA, USA) or anti-rabbitIgG secondary antibodies conjugated to HRP (catalog no. 7074; 1:1000; Cell Signaling Technology, Inc.) for 1 h in room temperature. Protein bands were detected with chemiluminescent substrate (Thermo Fisher Scientific, Inc., Waltham, MA, USA) and then measured using ImageJ software (version 1.37; National Institute of Health) and were normalized to GAPDH.

### 4.7. Statistical Analysis

One-way ANOVA identified significant differences with Tukey’s test for multiple comparisons with GraphPad Prism v4.0 (GraphPad Software, San Diego, CA, USA). Values are expressed as the mean ± standard error of at least three separate experiments. *p* < 0.05 was considered to indicate a statistically significant difference.

## 5. Conclusions

This study investigated the expression distribution of amylin in the rat brain treated with steroid hormones and in mouse neuroblastoma N2a. Amylin protein is expressed in the cerebral cortex neurons and dopaminergic neurons of the VTA of the immature rat brain. It was confirmed for the first time that amylin protein is strongly expressed in the cytoplasm of N2a cells. Additionally, P4 and Dex influence the expression of amylin protein in the rat brain and N2a cells.

## Data Availability

The data presented in this study are available on request from the corresponding author.
